# Advances in Vaccine Development

**DOI:** 10.3390/vaccines9091036

**Published:** 2021-09-18

**Authors:** Ralph A. Tripp

**Affiliations:** Department of Infectious Diseases, College of Veterinary Medicine, University of Georgia, Athens, GA 30602, USA; ratripp@uga.edu

The Special Issue titled “Advances in Vaccine Development” contains articles, reviews, and a perspective on advances in vaccine delivery and expression, nanovaccines, epitopes, proteins and adjuvants, and new vaccine platforms. The Special Issue covers topics including viruses, bacteria, and parasites, as well as the development of vaccines, adjuvants, and bioinformatic approaches.

The opening article by Penkert et al. [1] centers on the lack of a standardized parvovirus B19 neutralization assay, which is needed for the development of a parvovirus B19 vaccine. To address this, a unique region of VP1 containing prominent targets of neutralizing antibodies was designed using a mutated VP1 fragment (VP1uAT), which yielded enzyme-linked immunoassay (ELISA) results that correlated with neutralization. In the following article, Dunagan et al. [2] examine the impact of influenza A virus (IAV) proteins on host immune responses, specifically two host shutoff proteins, PA-X and NS1, which antagonize the host innate immune response. Using recombinant A/California/04/2009 containing mutations affecting PA-X and NS1 activity, they show that viruses without shutoff activities induce the strongest T and B cell responses and that the shutoff of NS1 suppressed lymphocyte migration to the lungs, antibody production, and the generation of IAV-specific CD4+ and CD8+ T cells. An article by Radwanska et al. [3] shows, in a DTP vaccine model with *trypanosome brucei brucei* infection, that treatment provides full recovery from infection but fails to restore an anti-Bordetella pertussis vaccine recall response and that this is related to the destruction of the host’s adaptive immune system by the trypanosome infection. It is unknown if the trypanosome infection causes permanent damage to the immune system or immunosuppression. This study is the first to show that trypanosome-inflicted immune damage is not restored by successful anti-parasite treatment. Finally, Chiozzini et al. [4] examine the intramuscular injection of the DNA vector vaccine platform using an array of viral products. The studies examine the efficiency of loading an HIV-1 Nef^mut^-based vaccine platform into extracellular vesicles (EVs). The findings show that when DNA vectors expressing Nef^mut^-based fusion proteins are intramuscularly injected in mice, significant quantities of the HIV-1 fusion proteins are packed into EVs. These engineered EVs are internalized by antigen-presenting cells, which cross-present the EV contents to activate antigen-specific CD8+ T cells. This study was aimed at increasing the vaccine platform safety profile by identifying the minimal part of Nef^mut^ retaining the EV-anchoring protein property. Importantly, Nef^mut^ C-terminal deletion did not affect the levels, quality, and diffusion of the antigen-specific CD8+ T cell immunity at distal sites. This suggests that the Nef^mut^-based technology is functional in inducing CD8+ T cell immunity against different (SARS-CoV-2) antigens.

Notable reviews follow the articles and are focused on different topics related to advances in vaccine development. They are grouped by topic and briefly summarized below. In the reviews related to SARS-CoV-2 or COVID-19, there is first a review by Rijkers et al. [5], which focuses on the antigen presentation of mRNA-based and virus-vectored SARS-CoV-2 vaccines; the mRNA vaccine composition and incorporation with lipid nanoparticles is examined. The novelty of mRNA vaccines is discussed, including vaccine cellular uptake, intracellular routing, processing, and secretion of the viral protein. The importance of virus-neutralizing antibodies and CTL are noted with regard to the technology. In a review by Chatterjee et al. [6], the role of next-generation bioinformatic approaches for coronavirus vaccine discovery and development is presented. The review discusses the stages of the vaccine development and available vaccines against COVID-19, and reviews tools and databases that are available for the identification and analysis of SARS-CoV-2 including molecular docking, vaccine and drug discovery, and comparative genomics analysis to facilitate vaccine development activities. The detailed review by Mellet and Pepper discusses the progress of different phases of COVID-19 vaccine development and the potential challenges once a vaccine becomes available. The review uncovers features that make it unclear whether a vaccine will be able to completely eradicate COVID-19, and discusses vaccination methods and strategies that may be key to success. The COVID-19 pandemic continues to spread across the world despite efforts to curb the transmission of the virus. Early vaccine trials were established to evaluate safety but also to demonstrate some degree of immunogenicity, i.e., the ability to generate neutralizing anti-S protein antibodies. There are additional aspects of COVID-19 immunity that must be considered, including the distinction between disease and infection, which may be asymptomatic but still result in transmission. The review by Giurgea and Memoli highlights these issues facing SARS-CoV-2 vaccinology.

Other viral vaccine-related reviews focused on barriers to human norovirus (HuNoV) vaccine development and the lack of robust and reproducible infection models. The review by Zhang et al. [7] considers advances in HuNoV vaccine research and summarizes the advances of the HuNoV cultivation system and HuNoV vaccine research, including the challenges and perspectives in future HuNoV vaccine development. The review highlights several HuNoV vaccines that have been designed and tested in preclinical models, and helps develop a better understanding of the features of protective immunity required to protect against HuNoV infection. It is known that elderly individuals are at an increased risk of developing severe symptoms including pneumonia and bronchiolitis following RSV infection. The diminished immune responsiveness by older individuals makes it more difficult to induce a robust immune response following vaccination. The review by Stephens and Varga [8] examines the current literature on RSV vaccines for the elderly and discusses factors to consider for generating the most efficacious vaccine formulation. The review considers the hurdles to developing a safe and effective RSV vaccine for use in the elderly and discusses the vaccine candidates currently being tested in this highly susceptible population. The review by Biagi et al. [9] examines the current state and challenges in developing respiratory syncytial virus (RSV) vaccines, summarizing the current state of RSV vaccine research and its implications for clinical practice while focusing on the characteristics of the vaccines that reached the clinical stage of development. Importantly, it is noted that while waiting for a safe and effective vaccine, therapeutics must be addressed for selected groups of high-risk populations. A growing number of human cytomegalovirus (hCMV) vaccine candidates have been developed, but none have been licensed due to many intrinsic features of hCMV that are challenging for vaccine design. In the review by Scarpini et al. [10], the aim was to assess the status of hCMV vaccines undergoing clinical trials, understand the barriers limiting vaccine development, and predict an effective multi-epitope vaccine constructed from PC, gB, and pp65 epitopes. Hepatitis C virus (HCV) is a major causative agent of acute and chronic hepatitis, and an efficient prophylactic vaccine remains unavailable. In the review by Velázquez–Moctezuma et al. [11], the understanding of HCV-specific neutralizing antibodies, antibody escape studies, and escape mutations is discussed. The review considers antibody escape and permissive animal models, and the selection of vaccine antigens for the development of HCV vaccine candidates capable of inducing enduring cross-genotype protection from HCV. In a thorough review by Müller et al. [12], the understanding of anti-rabbit hemorrhagic disease virus (RHDV) resistance and immunity, RHDV vaccination, and the establishment of rationally based RHDV vaccines is discussed. They propose the development of an RHDV vaccine that drives only B-cell-mediated immunity, but also adapted cellular immunity, using rationally based RHDV vaccines. 

Bacterial vaccine-related reviews were the subject of a review by Sousa et al. Their review, [13], is on immunization and immunotherapy approaches against bacterial infections, specifically pseudomonas aeruginosa and the *Burkholderia cepacia* complex (Bcc). In addition, ongoing clinical trials against pseudomonas aeruginosa infections are reviewed, and novel bacterial targets for the development of immunization and immunotherapy strategies against pseudomonas aeruginosa and Bcc infections are considered. *Acinetobacter baumannii* is an opportunistic Gram-negative coccobacillus that exhibits intrinsic resistance to routinely prescribed antibiotics. The development of multidrug-resistant *A. baumannii* strains are now prevalent worldwide. In the review by Ma and McClean, the current knowledge on virulence factors relating to *A. baumannii*–host interactions are summarized, as well as its implications in vaccine design. In the review by Chu and Quan, the current advances in *Toxoplasma gondii* vaccine strategies are summarized and the challenges hindering vaccine development clarified. The recent progresses in the development of toxoplasmosis vaccines using DNA, protein, nanoparticles, live-attenuated, and carbohydrate vaccine platforms are addressed, as well as some accomplishments in animal models. The review by Whitlow et al. [14] considers current vaccination strategies and offers directions for the development of new TB vaccines while highlighting recent findings. The discussion focuses on efforts for improved TB vaccines that may lead to new licensed vaccines capable of replacing/supplementing BCG and conferring therapeutic value in patients with active/latent TB. Vaccine candidates in the pipeline at different stages of clinical trials are noted, and the progress made in the development of improved vaccines evaluated. The interactions between microbiota and vaccination remains preliminary, and the available information suggests that the immune modulation by the microbiota may influence the vaccination response. In the review by Cianci et al. [15], the interplay between immunosenescence and microbiota in the efficacy of vaccines is examined. The role of gut microbiota in modulating immunity is discussed for older populations in particular, and a role of microbiota in boosting the response to vaccinations is proposed.

Other reviews on topics related to advances in vaccine development include the review by Cappellano et al. [16]; examining nano-microparticle platforms for the development of next-generation vaccines, they report on vaccine development using engineered nanoparticles (NPs) as vaccine delivery platforms. The review provides an overview of liposomes, EVs, and PLGA-NPs as carriers for vaccines against viral infections. Proteins, plasmid DNA, and mRNA are considered as components of NPs. The advantages of next-generation nano/microparticle-based vaccines are considered in comparison to traditional vaccines because they can induce a more potent CD8+ T cell response and are ideal carriers for peptides, adjuvants, mRNA, and DNA in the development of the next-generation vaccines. The review by Pippa et al. [17] examines recent advances and viewpoints on polymer-based nanovaccines, summarizing the properties, characteristics, added value, and limitations. Polymer-based nanovaccines can be used as delivery vehicles in various forms and morphologies for mRNA, DNA, and/or adjuvants in vaccines. Several advantages of polymer-based nanovaccines are mentioned, including robust cellular immune responses, an increased secretion of cytokines, and increased levels of antibodies and antigen-specific antibodies. The production of subunit nanovaccines relies on the development of a vaccine delivery system that is safe and efficient at delivering antigens to the target site. In the review by Lu et al. [18], covalent conjugation strategies for grafting proteins or peptide antigens onto other molecules or nanoparticles to obtain subunit nanovaccines are scrutinized, as are the advantages of chemical conjugation in developing these vaccines. Rapaka and colleagues consider the use of adjuvants to drive T cell responses for next-generation infectious disease vaccines. They review recently studied adjuvants and their potential for antigen presenting cell and T cell programming during vaccination with an emphasis on what has been observed in humans. The roles of T helper cell function, T cell memory, and CD8+ T cell cytotoxicity are considered in optimal and long-lived immunity induced by vaccination. A mini-review by Walkowski et al. [19] covers intranasal vaccine delivery technology for respiratory tract disease applications with a particular emphasis on pneumococcal disease. Some important elements of disease progression and the application of intranasal delivery in the treatment of these diseases are discussed, including the impact upon the elderly population. The advantages of intranasal vaccine delivery are given for pneumococcal disease, and the review highlights ongoing approaches. A perspective by Clemen and Bekeschus discusses ROS cocktails as an adjuvant for personalized antitumor vaccination. A new technical approach is proposed to optimize immunization by mimicking the relevant biological process of the inflammatory microenvironment, namely the generation of reactive oxygen species (ROS). It is proposed that medical gas plasma jet technology is capable of providing multi-ROS cocktails that can be implemented to provide efficient autologous antitumor vaccination.

In a departure from reviews related to viruses, van Oosterwijk et al. [20] address the phenomena and immunology behind resistance to tick infestation by livestock and laboratory animals, the extent of tick countermeasures to host immune defenses, and the impact of genomics and proteomics on complex tick–host interactions. This review examines novel antigens emerging from the tick microbiome which may aid in the development of anti-tick vaccines and treatments that block transmission in human, companion, and domestic animals as well as wildlife. 

Altogether, the articles, reviews, and perspective in this Special Issue of “Advances in Vaccine Development” highlight advances in the delivery and expression of antigens, nanovaccines, proteins, and adjuvants that create the potential for new vaccine platforms. Strategic approaches for selecting vaccine antigens using bioinformatics and novel formulations or regimens expand the field of vaccine research.
